# A Conditioned Behavioral Paradigm for Assessing Onset and Lasting Tinnitus in Rats

**DOI:** 10.1371/journal.pone.0166346

**Published:** 2016-11-11

**Authors:** Edward Pace, Hao Luo, Michael Bobian, Ajay Panekkad, Xueguo Zhang, Huiming Zhang, Jinsheng Zhang

**Affiliations:** 1 Department of Otolaryngology-Head and Neck Surgery, Wayne State University School of Medicine, 4201 Saint Antoine, Detroit, Michigan 48201, United States of America; 2 Department of Electrical Engineering, Wayne State College of Engineering, 5050 Anthony Wayne Drive, Detroit, Michigan 48202, United States of America; 3 Department of Biological Sciences, University of Windsor, 401 Sunset Avenue, Windsor, Ontario, Canada; 4 Department of Communication Sciences & Disorders, Wayne State University College of Liberal Arts & Sciences, 60 Farnsworth St., Detroit, Michigan 48202, United States of America; Universidad de Salamanca, SPAIN

## Abstract

Numerous behavioral paradigms have been developed to assess tinnitus-like behavior in animals. Nevertheless, they are often limited by prolonged training requirements, as well as an inability to simultaneously assess onset and lasting tinnitus behavior, tinnitus pitch or duration, or tinnitus presence without grouping data from multiple animals or testing sessions. To enhance behavioral testing of tinnitus, we developed a conditioned licking suppression paradigm to determine the pitch(s) of both onset and lasting tinnitus-like behavior within individual animals. Rats learned to lick water during broadband or narrowband noises, and to suppress licking to avoid footshocks during silence. After noise exposure, rats significantly increased licking during silent trials, suggesting onset tinnitus-like behavior. Lasting tinnitus-behavior, however, was exhibited in about half of noise-exposed rats through 7 weeks post-exposure tested. Licking activity during narrowband sound trials remained unchanged following noise exposure, while ABR hearing thresholds fully recovered and were comparable between tinnitus^(+)^ and tinnitus^(-)^ rats. To assess another tinnitus inducer, rats were injected with sodium salicylate. They demonstrated high pitch tinnitus-like behavior, but later recovered by 5 days post-injection. Further control studies showed that 1): sham noise-exposed rats tested with footshock did not exhibit tinnitus-like behavior, and 2): noise-exposed or sham rats tested without footshocks showed no fundamental changes in behavior compared to those tested with shocks. Together, these results demonstrate that this paradigm can efficiently test the development of noise- and salicylate-induced tinnitus behavior. The ability to assess tinnitus individually, over time, and without averaging data enables us to realistically address tinnitus in a clinically relevant way. Thus, we believe that this optimized behavioral paradigm will facilitate investigations into the mechanisms of tinnitus and development of effective treatments.

## Introduction

Tinnitus, a phantom auditory perception that occurs in the absence of external acoustic stimulation, is a prevalent health condition. It affects approximately 50 million Americans [1, 2], and can impair everyday life [3–7], costing an estimated 2 billion dollars for U.S. veterans alone [8]. This, in addition to the rising elderly population and the correlation between tinnitus and aging, underlines the need for effective tinnitus treatment. Currently, however, the mechanisms subserving tinnitus perception are poorly understood and tinnitus treatments are not consistently effective. A vital step to advance progress in these two arenas is to clearly understand the underlying mechanisms of tinnitus as related to clinical diagnosis and treatment. Achieving this step requires in-depth investigations using animal models. Thus, the development of reliable paradigms for testing tinnitus-related behaviors is urgently needed.

One of the most frequently-used paradigms for assessing tinnitus-like behavior in animals is the gap-detection paradigm [9, 10]. Gap-detection provokes an unconditioned reduction in acoustic startle reflex magnitude, except when the silent gap is obscured by other factors, such as presumed tinnitus perception. As such, only acclimation training is required to establish baseline behavioral performance in animals, while food/water deprivation, foot-shocking, and prolonged behavioral conditioning are all avoided. Gap-detection has been used to assess onset and lasting tinnitus-like behavior from a variety of inducers and animals, and may provide characteristics of tinnitus-like behavior, including pitch, duration, and diagnosis of individual animals [10]. Recent studies, however, have raised questions about potential confounding factors, such as general startle reflex reduction following acoustic trauma [11–13] and the possibility that tinnitus may not necessarily impair gap-detection [14–18]. While this paradigm remains to be validated using rigorously controlled metrics in both animal and human studies, behavioral paradigms that utilize conditioning procedures should further be explored.

The first conditioned behavioral paradigm for assessing tinnitus-like behavior in animals was introduced nearly thirty years ago [19]. Many other paradigms based on conditioning procedures have since been developed [20–24] and can possess unique benefits, such as reduced experimental stress [22], simultaneous recording of neural activity [20], and identification of the laterality of tinnitus-like behavior [25, 26]. Despite these benefits, the efficiency of existing paradigms is often compromised by prolonged behavioral training requirements (1–2 months or longer), an inability to simultaneously assess features such as tinnitus pitch and chronicity, and the need to compile data from multiple animals or testing sessions [27].

We have developed a conditioned licking suppression paradigm with a number of strengths, such as a lack of prolonged training requirements, and the ability to determine tinnitus pitch, onset versus lasting status, and presence of tinnitus-like behavior within individual animals and testing sessions. Although other conditioned paradigms may have one or more of the aforementioned strengths, the advantage of our paradigm is that it simultaneously possesses several strengths. In our “optimized” paradigm, animals were trained to lick during sound presentation to receive water rewards and to suppress licking during silence to avoid footshocks. An exposure to a known tinnitus inducer such as noise trauma or sodium salicylate increased licking during silence, indicating tinnitus-like behavior. Overall, we believe that the establishment of this paradigm will help bolster investigations into the underlying mechanisms of tinnitus and development of effective treatment strategies.

## Materials and Methods

### Behavioral setup and preparation

**Fig 1:**
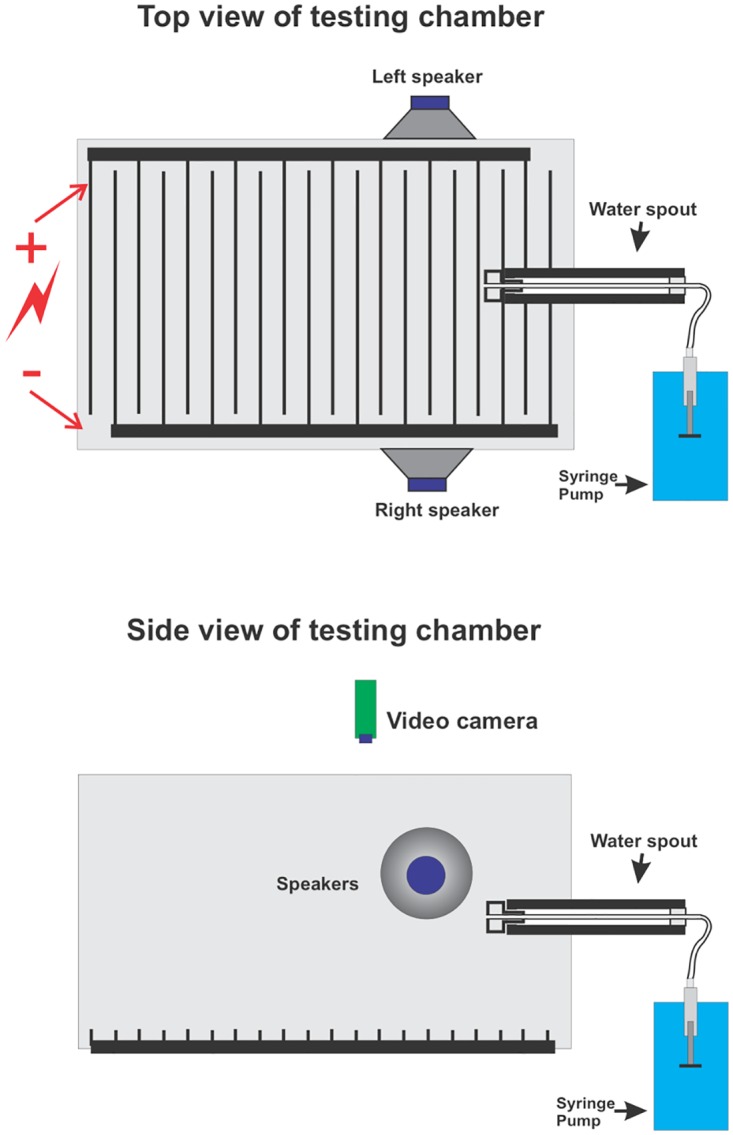
Top and side views of the behavioral testing chamber. The horizontal waterspout is located in the front of the wire mesh chamber and connected to a syringe pump for water delivery. Speakers were mounted to the chamber walls on both sides of the waterspout, so that sound could be presented bilaterally to the animal. Sounds levels and frequencies were calibrated using a microphone (ACO Pacific, Belmont, CA) and the chamber was tested to ensure that sound presentation did not vibrate the chamber. The stainless steel grid floor was electrified to deliver footshocks. Behavioral sessions were monitored with a USB camera placed above the testing chamber.

Behavioral training/testing was conducted with a rat in a custom-made, wire mesh chamber (27 x 8 x 14 cm^3^) (Fig 1) located inside a sound attenuation booth (Industrial Acoustic Co.). The rat could obtain a water reward by licking a custom-made horizontal waterspout, which was stationed at the front end of the chamber. When a rat licked the spout, it completed a mechanical circuit and triggered a water reward, which was delivered via an NE-1000 programmable syringe pump (New Era Pump Systems, Inc.) connected to the rear of the spout. Sounds were presented bilaterally from two electrostatic speakers (EC1 model, Tucker-Davis Technologies [TDT]). Mild electric foot-shocks were delivered through the stainless steel grid floor of the chamber using an isolated pulse stimulator (A-M Systems, Model 2100). The reaction of rats to the shock ranged from simply moving away from the spout to rapid backpedaling. The generation of sound waveforms, delivery of water rewards and footshocks, and collection of behavioral data were controlled by an RX6 multifunction processor- and RX7 stimulator-base station (TDT), as well as OpenEx software (TDT). A rat’s behavior was also observed with an overhead USB webcam (Webcam Pro 9000, Logitech).

Rats were water-deprived in their home cages and only obtained water during daily behavioral training/testing sessions. They were given pieces of apple following behavioral sessions to maintain hydration and 85–90% or more of ad lib body weight. Weights were measured prior to each session.

### Experiment 1

#### Animal subjects

Thirty adult male Sprague-Dawley rats were purchased from Envigo (formerly Harlan Laboratories, Indianapolis, IN, USA). The rats were ordered at 110 days of age with initial weights between 406g and 438g. All procedures were approved by the Institutional Animal Care and Use Committee at Wayne State University and were in accordance with the regulations of the Federal Animal Welfare Act.

#### Behavioral training—before tinnitus induction

During the first phase of training (training sessions 1–2), a rat was placed in the wire mesh chamber while a constant 60 dB SPL broadband noise (BBN) was presented. A rat received one water reward (30 μL) for each spout lick. A training session ended when the rat indicated satiety by not licking the spout for 3–4 consecutive minutes. Each phase one session lasted 15–30 minutes.

In the second phase of training (training sessions 3–10), a rat was placed in the mesh chamber while BBN were presented. A randomized sequence of BBN sound trials (200 total) and silent trials (50 total) were presented. Each BBN sound trial or silent trial was 8 s in duration. A rat only received one water reward for every third spout lick, to increase the reliance of licking behavior on sound presence versus water rewards alone. On day 3, a rat was not shocked if it licked during a silent trial. From days 4 through 10, however, a silent trial lick triggered a footshock (1 s duration). The lowest level of shock that could make an animal break its contact with the spout was selected for each rat (typically 0.25–0.75 mA), in order to minimize licking during silence while avoiding significant fear association with the spout. A rat was not shocked for licking during the first two seconds of a silent trial to provide enough time for it to withdraw from the spout. A rat never received more than 10 footshocks in a single training/testing session to prevent potential distress from frequent shocks. A rat was considered to have stable baseline behavior when it averaged ≤ 1 lick per silent trial for 4 consecutive testing sessions. This criterion was selected because once it was reached, rats did not exceed that rate or exhibit a significant decrease in silent trial licking. A full training session from this phase onward lasted approximately 33 minutes.

The third phase of training (training sessions 11–13) was similar to phase two except that each sound trial was comprised of a narrowband noise varying from 6–8, 10–12, 14–16, 22–24, or 30–32 kHz. Narrowband noises were introduced so that the pitch of tinnitus-like perception, if present, could be examined (see Data Analysis section for details). Each training session consisted of 40 trials of each narrowband noise (a total of 200 sound trials) and 50 silent trials. For each narrowband noise, the 40 sound trials were randomly interspersed by a total of 10 silent trials. Thus, a silent trial was categorized by the preceding narrowband sound trial (i.e. a “6–8 kHz” silent trial). A rat received no more than 2 shocks over the 10 silent trials associated with each narrowband noise category, for a maximum of 10 shocks per training session. A rat was considered to have stable baseline behavior during this phase when it averaged ≤ 1 lick per each category of silent trial.

**Fig 2:**
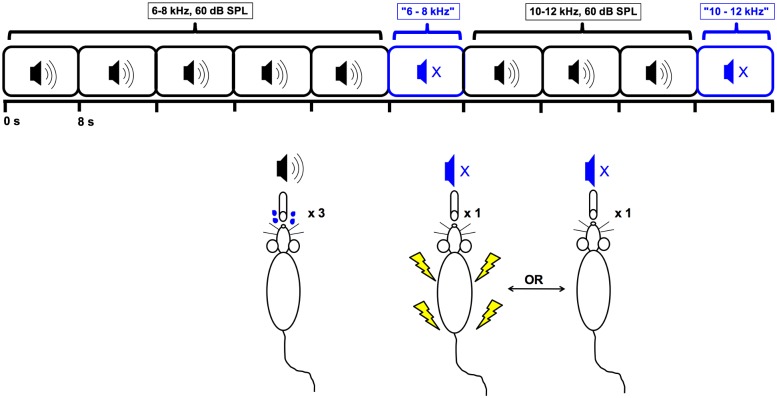
Illustrations showing phase 4 behavioral training during 6–8 and 10–12 kHz sound and silent trials. The “x 3” and “x 1” notations refer to the number of spout licks required to obtain a water reward and a variable shock, respectively.

In the fourth training phase (training sessions 14–16, Fig 2), sound presentations were the same as in the third phase but footshocks were applied for no more than 50% of silent trial licks (up to a maximum of 10 shocks). This partial shock feedback schedule was used to ensure that an animal would rely primarily on detection of silence rather than shock signals for cessation of licking. Stable baseline behavior was considered when a rat averaged ≤1 spout lick per silent trial.

The same procedures used in phase 4 behavioral training were used for testing tinnitus behavior following exposure to intense noise, or injection of sodium salicylate or physiological saline.

#### Recording of auditory brainstem responses (ABRs)

ABRs were recorded before noise or sham noise exposure, immediately after exposure, and 7 weeks after exposure to evaluate hearing thresholds. A rat was anesthetized using a mixture of air (0.4 L/min) and isoflurane (2–3%, v/v) and placed in a prone position with its head fixed to a stereotaxic frame. Body temperature was maintained using a heating blanket connected to a thermostatic controller (Harvard Instruments, Holliston, MA, USA). Acoustic stimuli included clicks (0.1 ms) or tone bursts (10 ms) presented at 8, 12, 16, 24, or 32 kHz, and were delivered through a speaker tube inserted into the external auditory canal. Three subcutaneous platinum-coated tungsten electrodes were used to record ABR waveforms, with the reference electrode located below the pinna ipsilateral to the speaker tube, the grounding electrode located below the contralateral pinna, and the recording electrode located at the vertex. Evoked potentials were bandpass-filtered at 300–3000 Hz, notch-filtered at 60 Hz, and averaged 300 and 400 times for clicks and tone-bursts, respectively. Data were recorded using BioSigRP^®^ and SigGenRP^®^ software (TDT) installed on an IBM computer connected to a System 3 TDT workstation.

#### Noise exposure

After stable baseline behavioral data and ABR data were acquired, rats were binaurally exposed to an intense band noise (8–16 kHz, 105 dB SPL, 2 hours) while they were awake [28]. Briefly, three to four rats were placed in a 44 × 23 × 22 cm polycarbonate cage with corncob bedding and were exposed to the intense noise together. The intense noise was presented from a TW67 speaker (Pyramid Car Audio, Brooklyn, N.Y.) placed facedown on top of the cage. Sound waveforms were generated digitally using an RX6 multifunction processor and amplified through an RA 300 amplifier (Alesis, Cumberland, RI). The noise exposure procedure was controlled by a custom-made OpenEx program. The speaker was calibrated at the center of the cage using a sound pressure level meter (Bruel & Kjar, BZ-7100). One week later, rats were exposed to a second intense noise (8–16 kHz, 110 dB SPL, 2 hours). Two exposures were used to facilitate tinnitus induction while avoiding permanent hearing threshold shifts. The second exposure also increased clinical relevance of our study, as many individuals in human populations have experienced more than one acoustic trauma incident. Exposing animals while they were awake added further clinical significance and avoided the protective effects of anesthesia [29–31]. Post-exposure behavioral testing began 30 minutes after the conclusion of noise exposure (post-exposure day 0). For control animals, sham exposure was conducted using the same procedures except that the intense noise was not delivered.

#### Sodium salicylate administration

To broaden the applicability of our behavioral paradigm, we also assessed salicylate-induced tinnitus. Eight weeks following the second intense noise exposure, sodium salicylate (350 mg/kg, 50 mg/ml, Sigma) or saline control solution (5 mL/kg) was injected intraperitoneally to animals that did not exhibit tinnitus-like behavior during the previous 3 weeks. The dosage has been reported to reliably induce tinnitus in animals. The day before injections, animals were behaviorally tested to verify their lack of tinnitus-like behavior. Behavioral tests were conducted again 3 hours following injections.

#### Behavioral testing procedure after induction of noise and tonal tinnitus

Following noise/sham exposure or sodium salicylate administration, behavioral testing was conducted using the same parameters as described in phase 4. Post-noise-exposure testing was conducted until both tinnitus positive behavior and tinnitus negative behavior stabilized within individual rats (see Results). Post-salicylate-injection testing was conducted to observe the occurrence and then disappearance of tinnitus-like behavior.

### Experiment 2

To examine whether electrical shock exerted any effects on the validity of the behavioral data collected above, we conducted supplementary experiments. In these experiments, we first behaviorally trained animals as described in Experiment 1. Next, for all of the testing sessions conducted after noise or sham noise exposure, rats were no longer shocked for licking the spout during silent trials. This experiment was conducted in 20 adult male Sprague-Dawley rats. The rats were ordered at 110 days of age with initial weights between 398g and 427g. These rats were water-deprived and underwent conditioned licking suppression training using the same procedures as those described in Experiment 1. Following behavioral training, rats were divided into one of four groups, with five rats per group. The first and second groups received one noise exposure (8–16 kHz, 115 dB SPL, 2 hours) or sham noise exposure, respectively. The sham exposure procedure was identical to the noise exposure procedure except that no intense noise was delivered. We did not add a second noise exposure one week later, since we were not sure if waiting a week between the last behavioral training session and the second exposure would affect conditioned licking suppression. The first and second groups were tested for tinnitus-like behavior at 1, 2, and 4 weeks following the exposure. The third and fourth groups also underwent noise exposure or sham noise exposure, respectively, but they were tested at 4 and 8 weeks post-exposure. The behavioral procedure used for post-exposure testing was identical to the aforementioned phase four of baseline training except that foot-shocks were never administered for silent trial licks.

#### Data analysis: Experiments 1 and 2

Behavioral data: Behavioral data were analyzed offline using a custom Matlab script (MathWorks, Nattick, MA, USA), which extracted data from OpenEx data tanks to Excel software (Microsoft, Redmond, WA, USA). During a behavioral session, if a rat did not lick the spout during a sound trial, this trial and any consecutive trials (sound or silent) where the rat did not lick the spout were excluded from analysis. This prevented the arbitrary lowering of licking rates by other behaviors, such as grooming and exploring. Afterwards, the average number of licks per category of sound trial and silent trial, respectively, was calculated for each rat in each behavioral session. Prior to noise exposure, each rat averaged ≤ 1 lick per each category of silent trial from training session 13 through session 16. Furthermore, no statistically significant changes in licking rate was found within this time frame (see Results). Therefore, in Experiment 1 a rat was considered to have exhibited tinnitus-like behavior if it exceeded this licking rate following noise exposure or salicylate injections. In Experiment 2, both sham-exposed and noise-exposed rats increased silent trial licking during no shock testing. Therefore, noise-exposed rats were considered to exhibit tinnitus-like behavior when their silent trial licking rates exceeded the range of sham-exposed rats’ licking rates.

ABR data: ABR hearing thresholds were considered to be the lowest sound intensity at which a distinct portion of the ABR waveform was visible. Thresholds were determined for pre-exposure, post-exposure day 0, and post-exposure week 7 recordings.

Statistics. Mixed-model ANOVAs were used to assess behavioral and ABR data in Experiment 1. Greenhouse-Geisser corrections were used when Mauchly’s test of sphericity was violated. Significant results were followed by post-hoc Bonferroni tests where appropriate. P < 0.05 was considered significant. Statistical analysis was performed with IBM SPSS 21.0 software (Chicago, IL).

## Results

### Experiment 1

#### Baseline behavioral training before noise exposure or salicylate administration—silent trials

**Fig 3:**
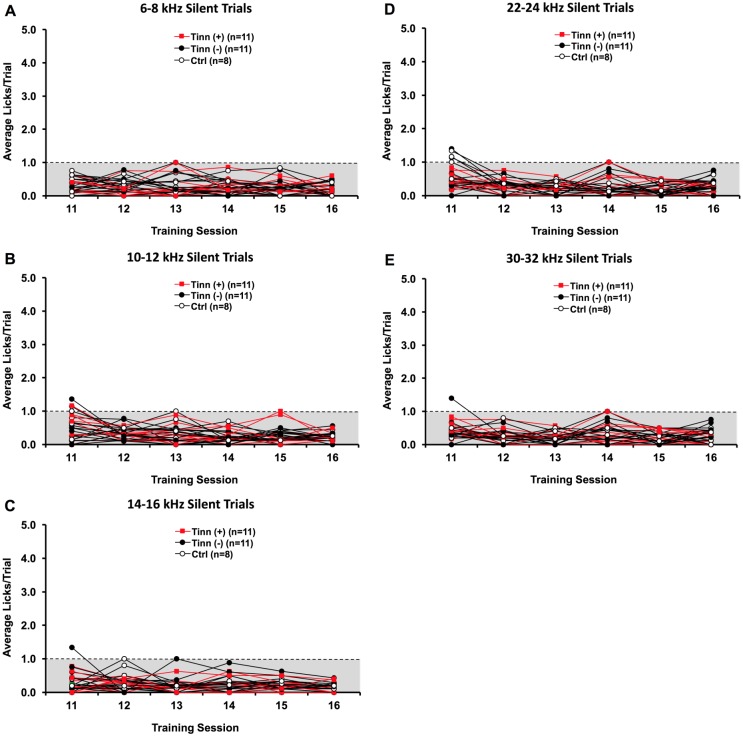
Baseline rates of licking during silent trials (training sessions 11–16). Tinnitus^**(+)**^ refers to rats that later exceeded 1 lick/trial for one or more silent trial categories over weeks 5 through 7 week following noise exposure; tinnitus^**(-)**^ refers to noise-exposed rats that did not meet that criteria. A silent trial category was determined by the narrowband sound trial that preceded it (i.e. 6–8 kHz, 10–12 kHz, 14–16 kHz, 22–24 kHz, 30–32 kHz) (A-E). From baseline test sessions 13–16, all rats exhibited stable baseline behavior (≤ 1 average lick/trial) for every silent trial category, as indicated by the dashed line and shaded area. There were no significant differences between tinnitus^(+)^, tinnitus^(-)^, or control (ctrl) rats in baseline silent trial licking.

In the initial training phases, rats were conditioned to lick during the presentation of BBN sound and to suppress licking during silence. Rats averaged as high as 5 licks per silent trial during the beginning of phase 2 training. By training session 6, however, all rats averaged ≤ 1 lick per silent trial and maintained this rate through the 10^th^ training session. In training phase 3 (sessions 11–16), a sound trial consisted of one of five narrowband noises, and silent trials were categorized according to the preceding narrowband noise. A few rats exceeded 1 lick per silent trial during training sessions 11 and 12, but all rats averaged ≤ 1 lick per each silent trial category by session 13 and maintained this rate between sessions 14 to 16 (Fig 3A–3E). There were no statistically significant differences in licking rate across sessions 13 through 16 (p > 0.05; *post-hoc* Bonferroni), indicating that performance had stabilized. Since the highest licking rate was 1/trial once behavior had stabilized, a rat that committed > 1 lick per any silent trial category following intense noise exposure was considered to exhibit tinnitus-like behavior. We compared noise-exposed animals that later developed lasting tinnitus-like behavior (from post-exposure week 5 or earlier through week 7; tinnitus^(+)^), noise-exposed animals that did not show lasting tinnitus-like behavior (tinnitus^(-)^), and sham exposed control animals (ctrl). No significant interaction was found between group and test (*F*_*(5*.*387*,*10)*_ = 0.419, p = 0.846) when comparing groups over all testing sessions and silent trial categories. Furthermore, the rate of licking was not different between the three groups (*F*_*(2*,*27)*_ = 1.118, p = 0.342) when comparing across all sessions and silent trial categories. This indicates that there were no preexisting differences in baseline licking suppression between rats from any group.

Once footshocks were introduced, they were initially delivered to all rats at 0.25 mA. We sought to use the lowest amplitude possible to break a rat’s contact with the spout. For rats that did not respond to 0.25 mA, the amplitude was increased up to but no greater than 0.75 mA. These amplitudes were maintained throughout the remainder of experiments.

#### Baseline behavioral training before noise exposure or salicylate administration—sound trials

**Fig 4:**
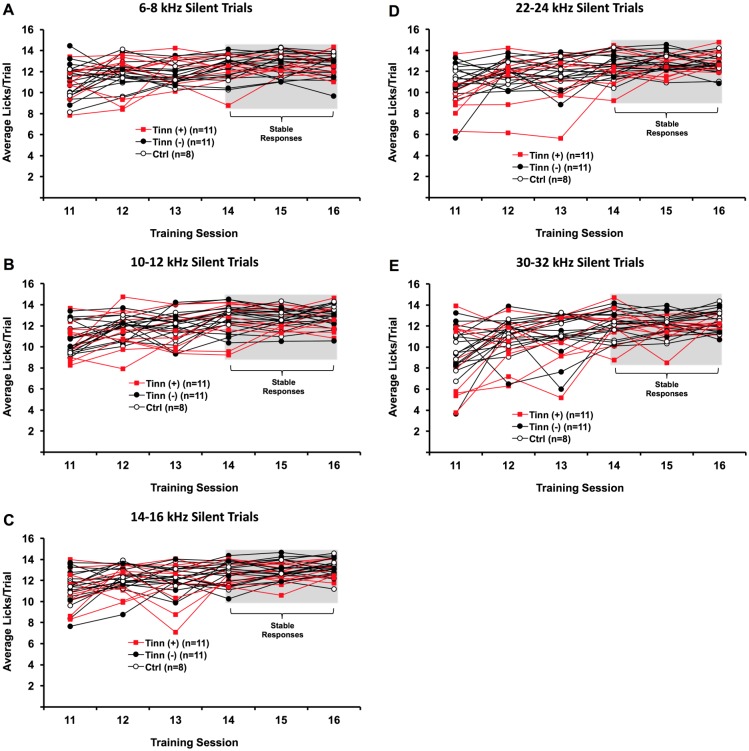
Baseline licking rates of eventual tinnitus^(+)^, tinnitus^(-)^, and control (ctrl) rats during different narrowband sound trials (A-E). Tinnitus^**(+)**^ refers to rats that later exceeded 1 lick/trial for one or more silent trial categories over weeks 5 through 7 weeks following noise exposure; tinnitus^**(-)**^ refers to noise-exposed rats that did not meet that criteria. For all rats, licking rates did not significantly change between training sessions 14 through 16. These rates were comparable between all groups of rats.

Average licking rates during sound trials were also assessed (Fig 4A–4E). No significant interactions were found between group and test (*F*_*(5*.*694*,*10)*_ = 0.620, p = 0.705) or between groups (*F*_*(2*,*27)*_ = 0.654, p = 0.528) when comparing across all testing sessions and silent trial categories. This indicates that there were no preexisting differences in baseline sound trial licking between eventual tinnitus^(+)^ and tinnitus^(-)^ rats, or controls. There were, however, significant differences in overall sound trial licking across training sessions (*F*_*(2*.*847*,*5)*_ = 30.009, p < 0.001). Specifically, rats licked more during sound trials in training sessions 14–16 than in sessions 11–13 (post-hoc Bonferroni; p < 0.05). This indicates that rats did not learn to lick robustly during narrowband sound trials up until training session 14. No significant differences in sound trial licking were found across training sessions 14–16 (post-hoc Bonferroni; p > 0.05). Baseline performance was thus considered to be stabilized by this time point. No further training was deemed necessary.

The total licks per training/testing session typically ranged between 1500 and 3000. The number of sound trials that had to be excluded for licking inactivity usually ranged between 10 to 30 per session.

#### Licking behaviors following intense noise exposure—silent trials

**Fig 5:**
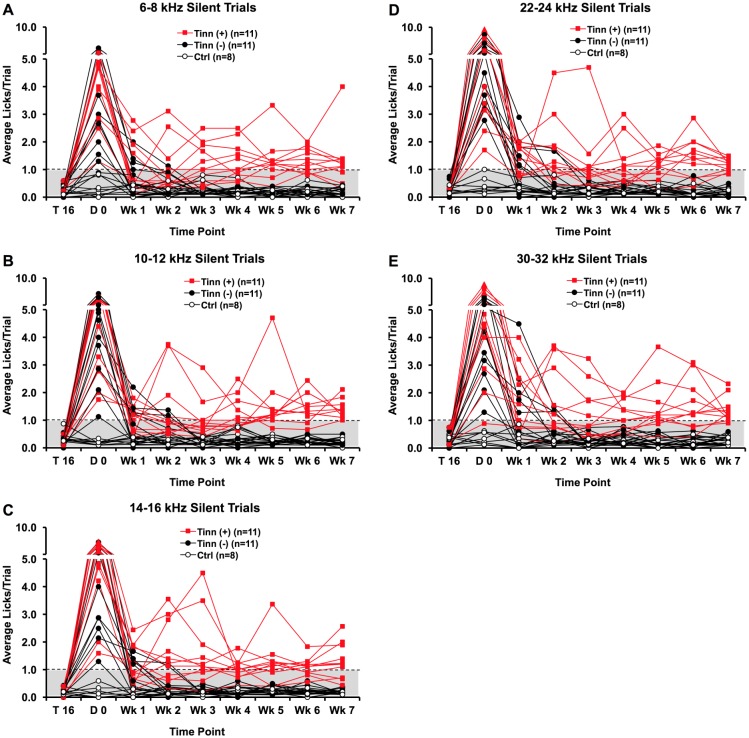
Licking rates over time for tinnitus^(+)^, tinnitus^(-)^, and control rats during silent trials (A-E). Tinnitus^**(+)**^ refers to rats that later exceeded 1 lick/trial for one or more silent trial categories over weeks 5 through 7 weeks following noise exposure; tinnitus^**(-)**^ refers to noise-exposed rats that did not meet that criteria. Tinnitus^(+)^ and tinnitus^(-)^ rats exceeded 1 lick per each silent trial category immediately after noise exposure (D 0), except for one rat for 6–8 and another rat for 30–32 kHz silent trials. Eleven rats exceeded 1 lick per silent trial for one or more silent trial categories over at least 5 to 7 weeks post-exposure (Wk 5 –Wk 7). These increases in silent trial licking were significant relative to the last baseline training session (T16). Control rats show no significant increases in licking over time. The dashed line and gray-shaded area indicate the ≤ 1-lick/trial threshold.

All 30 rats were subjected to two intense noise or sham exposures. They were tested behaviorally 30 minutes after the second exposure (day 0) and on a daily basis for 7 weeks (Fig 5A–5E). Twenty of the noise-exposed rats averaged greater than 1 lick/trial for all five silent trial categories (following 6–8, 10–12, 14–16, 22–24, and 30–32 kHz narrowband sound trials, respectively). Sixteen rats exceeded the 1-lick/trial rate for at least one silent trial category in post-exposure week 1. Twelve rats exceeded that rate in post-exposure week 2, and eight rats exceeded that rate in post-exposure week 3 and 4. Over weeks 5–7, however, 11 rats each consistently exceeded the 1 lick/trial rate for at least one silent trial category (tinnitus^(+)^), while the other 11 rats licked at the ≤ 1/trial rate for all silent trial categories (tinnitus^(-)^). Of the tinnitus^(+)^ rats, some consistently exceeded the 1-lick/trial rate from post-exposure day 0 to week 7, while others showed more variability until week 5 through week 7. No control rats exceeded the 1 lick/silent trial rate.

For group analysis, we compared licking rate changes over time individually for each of the three groups. Our results indicate that both of the eventual tinnitus^(+)^ (*F*_*(1*.*956*,*8)*_ = 28.098, p < 0.001) and tinnitus^(-)^ group rats (*F*_*(1*.*588*,*8)*_ = 60.624, p < 0.001) significantly increased silent trial licking immediately after exposure (post-exposure day 0) relative to baseline silent trial licking. Neither group showed an interaction between post-exposure time and silent trial category. Between 5–7 weeks post-exposure, the tinnitus^(+)^ group exhibited significantly increased silent trial licking rates (post-hoc Bonferroni; p < 0.05) relative to baseline, while the tinnitus^(-)^ group did not (post-hoc Bonferroni; p = 1). Control rats showed no change in silent trial licking when comparing post-exposure performance to baseline performance (*F*_*(2*.*511*,*8)*_ = 1.771, p = 0.135)

We also assessed licking rate as a function of silent trial category. Immediately after noise exposure, rats increased silent trial licking following all narrowband noises. This reflects an acute tinnitus-like behavior. By 7 weeks post-exposure, rats with chronic tinnitus-like behavior (tinnitus^(+)^) also increased silent trial licking following all narrowband noises. For some tinnitus^(+)^ rats, however, the increase was more predominant for some silent trial categories than for the others.

#### Licking behaviors following intense noise exposure—sound trials

**Fig 6:**
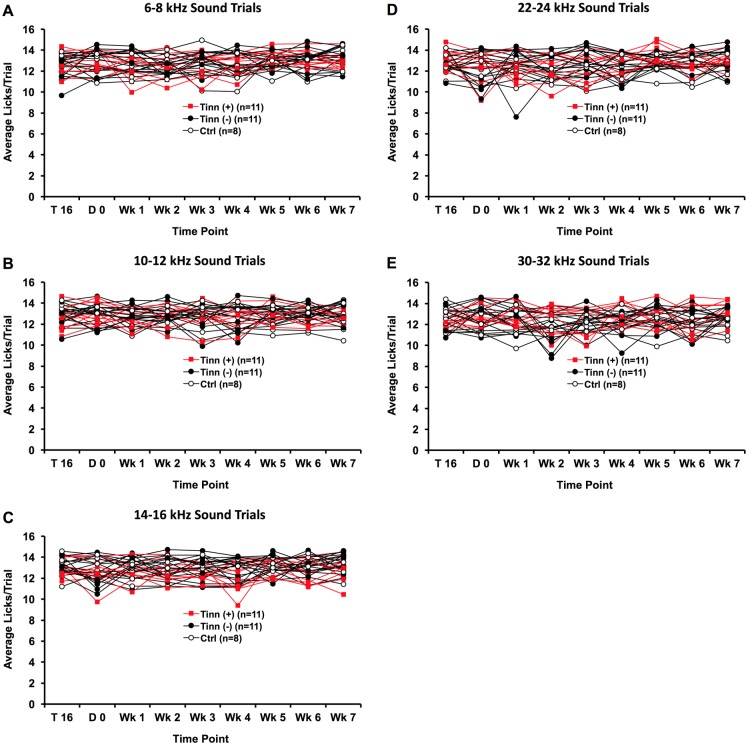
Licking rates over time for tinnitus^(+)^, tinnitus^(-)^, and control rats during narrowband sound trials (A-E). Tinnitus^**(+)**^ refers to rats that later exceeded 1 lick/trial for one or more silent trial categories over weeks 5 through 7 weeks following noise exposure; tinnitus^**(-)**^ refers to noise-exposed rats that did not meet that criteria. No significant changes in sound trial licking were observed for any group of rats.

The rate of licking in sound trials was assessed following intense noise exposure (Fig 6A–6E). We compared licking rate changes over time individually for each of the three groups. Neither tinnitus^(+)^ (*F*_*(3*,*8)*_ = 0.599, p = 0.750), tinnitus^(-)^ (*F*_*(3*,*8)*_ = 1.079, p = 0.529), nor control rats (*F*_*(3*,*8)*_ = 1.656, p = 0.13) exhibited significant changes in licking rates during any category of narrowband sound trial, relative to baseline sound trial licking. It appeared, therefore, that overall licking activity and responsivity to sound during post-exposure testing sessions were comparable to baseline levels.

#### Licking behaviors following salicylate or saline administration

**Fig 7:**
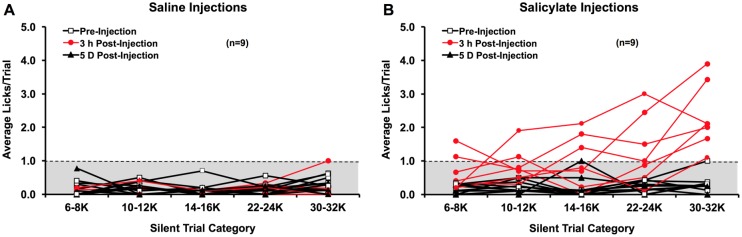
Licking rates during silent trials prior to injections (post-exposure week 8), as well as 3 hours and 5 days following saline (A) or salicylate (B) injections. After saline injections, no changes in licking rate were observed. Three hours following salicylate injections, however, all nine animals increased licking behavior during silent trials, though most robustly following high-frequency narrowband sound trials. The licking rates during silent trials returned to pre-injection levels by 5 days post-injection.

Eight weeks after the second noise exposure, nine of the tinnitus^(-)^ rats were intraperitoneally injected with saline (Fig 7A) and one week later with salicylate (Fig 7B) to test the effect on licking behavior. At both 3 hours and 5 days following saline injections, all rats maintained silent trial licking averages of ≤ 1. This was supported by group-wise analysis. Here, we found no significant differences in licking between time points across all silent trial categories (*F*_*(2*,*7)*_ = 2.934, p = 0.119), or interactions between time point and silent trial category (*F*_*(2*.*886*,*8)*_ = 0.409, p = 0.741). Therefore, rats maintained tinnitus^(-)^ behavior and were unaffected by the saline injections.

Three hours following salicylate injections, however, all rats exceeded an average of 1 lick for at least one silent trial category (*F*_*(1*.*166*,*2)*_ = 49.858, p < 0.001). Licking rates increased more during silent trials preceded by high-frequency narrowband sounds than those preceded by low-frequency narrowband sounds. This was in part reflected by a significant interaction between time and silent trial category (*F*_*(2*.*979*,*8)*_ = 4.308, p = 0.015). Specifically, licking significantly increased during all silent trials except for those preceded by 6–8 kHz narrowband sound trials (p = 0.068). All silent trial licking rates decreased to ≤ 1 by post-injection day 5 and were comparable to pre-injection rates, as supported by group-wise analysis (p = 0.887).

#### Auditory brainstem responses (ABRs)

**Fig 8:**
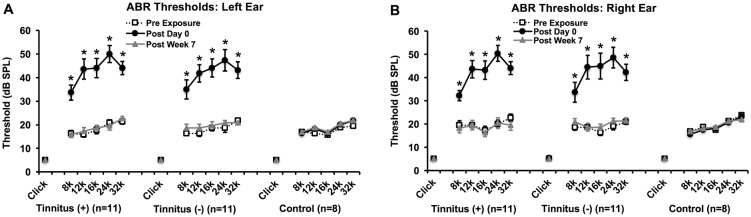
ABR hearing thresholds for tinnitus^(+)^, tinnitus^(-)^, and control rats prior to intense noise or sham exposure, and on post-exposure day 0 and post-exposure week 7. Thresholds were elevated across all frequencies in tinnitus^(+)^ and tinnitus^(-)^ rats at post-exposure day 0, revealing immediate and significant hearing loss. By post-exposure week 7, however, thresholds recovered to pre-exposure levels. At all time points, thresholds were similar between tinnitus^(+)^ and tinnitus^(-)^ rats. There were no threshold elevations in control rats. Error bars represent standard error of the mean.

ABRs were used to evaluate hearing thresholds prior to an intense noise exposure, as well as on day 0 and week 7 following the second exposure (Fig 8A and 8B). We found that noise exposure significantly affected hearing thresholds (*F*_*(1*.*150*,*2)*_ = 72.737, p < 0.001). Specifically, thresholds significantly increased on post-exposure day 0 (p < 0.001) but recovered by post-exposure week 7 (p = 1). There were no significant interactions between group, time, or frequency. For control rats, we observed no significant changes in hearing thresholds over time (*F*_*(1*.*561*,*2)*_ = 0.425, p = 0.616). There were also no overall differences between rats with lasting tinnitus^(+)^ behavior and tinnitus^(-)^ behavior (*F*_*(1*,*20)*_ = 0.002, p = 0.967). Thus, there were no permanent hearing threshold shifts or lasting differences between the experimental groups.

### Experiment 2

#### One-, two-, and four-weeks after noise-exposure or sham-exposure

In 12 rats, behavioral tests without shocks were conducted following either noise exposure or sham noise exposure. Overall, rats tested without shock feedback exhibited higher silent trial licking rates, with all rats exceeding the 1 lick/trial rate for at least one silent trial category. However, we found that even without shock feedback, noise-exposed rats licked significantly more than sham-exposed rats (*F*_*(1*,*8)*_ = 28.451, p < 0.001). There was no significant interaction between group and time (*F*_*(2*,*7)*_ = 0.8, p = 0.487).

**Fig 9:**
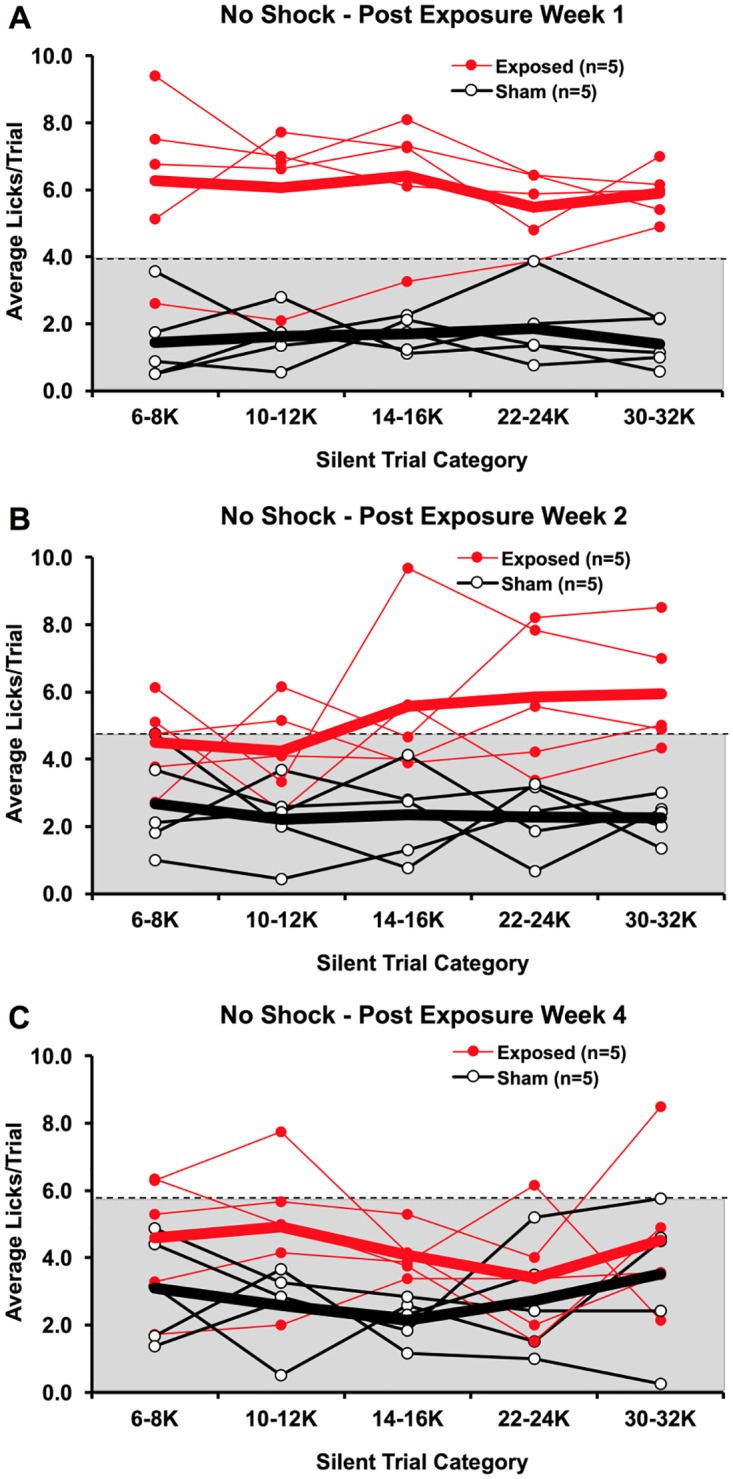
Noise-exposed (Exposed) and sham-exposed (Sham) rats tested without footshocks at 1, 2, and 4 weeks post-noise-exposure. Overall, exposed rats had higher silent trial licking rates compared to sham rats across a broad range of silent trial categories, especially at 1 and 2 weeks post-exposure. The higher licking rates are suggestive of tinnitus-like behavior. In exposed rats, the rate of silent trial licking, as well as the number of rats that exhibited elevated rates across a range of silent trial categories, tended to decrease over time. In sham-exposed rats, the rate of silent trial licking only slightly increased over time.

One week following exposure, all five of the noise-exposed rats exceeded 3.875 licks, which was the uppermost range of the sham-exposed rats. (Fig 9A). Four of the noise-exposed rats exceeded this range at all silent trial categories, while one noise-exposed rat only exceeded the range for 30–32 kHz silent trials. The latter rat may have exhibited tonal tinnitus-like behavior.

Two weeks following exposure, all five noise-exposed rats exceeded the sham-exposed licking range for at least one silent trial category (Fig 9B). However, the difference between the two groups of rats was not large as at one week following exposure, potentially indicating a reduction in tinnitus loudness or in sham rats’ conditioned licking suppression conditioning. Noise-exposed rats did not exceed the licking range of sham rats as broadly across silent trial categories, compared to one week post-exposure.

Four weeks following exposure, only 3 noise-exposed rats exceeded the licking range of sham-exposed rats (Fig 9C) at various silent trial categories. Like two weeks post-exposure (Fig 9B), the separation of noise-exposed and sham-exposed groups became smaller, also possibly due to reduced tinnitus loudness or reduced licking suppression. Overall, noise-exposed rats further decreased silent trial licking compared to the previous weeks, often averaging less than 4 silent trial licks.

#### Four- and eight-weeks after noise-exposure or sham-exposure

**Fig 10:**
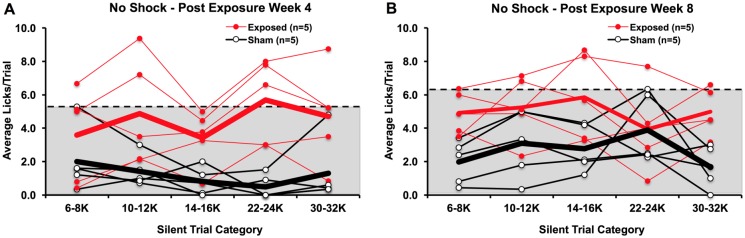
Noise-exposed (Exposed) and sham-exposed (Sham) rats tested without footshocks at four weeks (A) and eight weeks (B) following exposure. The dotted line and shading indicate the silent trial licking range of sham-exposed rats. A few of the noise-exposed rats exhibited silent trial licking rates higher than those of sham-exposed rats at both four and eight weeks post-exposure. This is suggestive of tinnitus-like behavior.

Behavioral testing without shocks was also conducted in two additional groups of animals at four and eight weeks following noise exposure or sham noise exposure. While we did observe a significant interaction between group and time (*F*_*(1*,*8)*_ = 7.753, p = 0.025), noise-exposed rats still committed significantly more licks than sham rats during each of the four and eight week testing points (p < 0.05). We found that 1 noise-exposed rat exceeded the silent trial licking range of sham-exposed rats across nearly all silent trial categories (Fig 10A), while another 2 noise-exposed rats only exceeded the range at 1 or 2 silent trial categories. Yet another 2 noise-exposed rats, however, did not exhibit licking rates above of the range of sham-exposed rats. These results were maintained at the eight-week time point (Fig 10B).

## Discussion

In these experiments, we sought to develop an optimized behavioral paradigm that is capable of efficiently assessing tinnitus-like behavior in animals. Specifically, we set out to establish a paradigm that did not require very long training periods (i.e. 1–2 months), that could determine onset tinnitus, lasting tinnitus, and tinnitus pitch, and that could determine tinnitus in individual animals over time and without averaging data. These capabilities would enable us to realistically address tinnitus in a realistic and clinically relevant manner. We believe that such optimized behavioral testing can significantly benefit the ongoing endeavors towards delineating the mechanisms underlying tinnitus, and developing effectiveness treatments.

### Evidence of tinnitus-like behavior

In Experiment 1, we demonstrated that immediately following intense noise-exposure or salicylate injections, all rats exceeded the 1 lick/silent trial rate that was the upward limit established during stable behavioral performance (training sessions 13–16; Fig 3). Furthermore, their post-exposure or post-injection licking rates were significantly higher compared to their baseline or pre-injection licking rates. In other words, rats were essentially acting against their conditioned licking suppression, presumably because they perceived sound instead of silence and were attempting to obtain water rewards. Therefore, rats that exceeded the 1 lick/silent trial rate were considered to exhibit tinnitus-like behavior. This criterion was further validated by the performance of control rats, which never exceeded the 1 lick/silent trial rate throughout the 7 weeks tested following sham exposure. The behavioral measurement and evidence of tinnitus-like behavior is similar to previous conditioned licking studies conducted within a few hours and days after acoustic trauma [23, 32], as well as onset tinnitus following sound exposure in humans [33, 34].

From weeks 5 to 7 following noise exposure, about half of our rats reliably exhibited tinnitus-like behavior whereas the other half did not. Between this time point and post-exposure day 0 (the day of exposure), some of the noise-exposed rats maintained tinnitus-like behavior while others exhibited remission of tinnitus-like behavior, or remission followed by reemergence. Overall, the variability in tinnitus behavioral development over time and between animals is in line with other studies [28, 32, 35–40]. This reflects the variable nature of tinnitus manifestation that has been documented in humans [41]. The fact that silent trial licking rates even varied within the tinnitus^(+)^ group and over time may be due to changes in tinnitus loudness or sensitivity, though this needs further investigation.

In addition to the presence of tinnitus-like behavior, we assessed rats’ behavior for evidence of tinnitus pitch. Rats that perceived tinnitus with a pitch were expected to increase silent trial licking specifically following narrowband sound(s) that resembled their tinnitus. We observed evidence of variable tinnitus pitch in some noise-exposed rats with lasting tinnitus-like behavior, as well as evidence of a high-frequency pitch in many salicylate-injected rats. Predominantly, we found that when noise-exposed rats exhibited tinnitus-like behavior, they increased licking during many or all silent trial categories. This finding may suggest evidence of noise-like tinnitus behavior. Diverse results have occurred in other studies, with lasting tonal tinnitus behavior [9, 21, 38, 42–45] and noise-like tinnitus behavior [28, 35] observed after noise-exposure. The high incidence of noise-like tinnitus behavior we observed may be due to the fact that rats were exposed to band noise (8–16 kHz), as opposed to more of a narrowband or pure tone exposure. For salicylate-induced tinnitus-like behavior, pitch has often manifested within 10 to 20 kHz [20, 46–53]. Others, however, have shown evidence of tinnitus pitch at up to 32 kHz and even BBN [54–58], matching the current study. Thus, it is evident that even salicylate-induced tinnitus-like behavior may present with a range of spectral features. Our finding that salicylate-induced tinnitus only manifested for a few days after injections and then disappeared is consistent across all of the aforementioned salicylate studies.

To help corroborate the evidence of tinnitus-like behavior, we assessed hearing thresholds with ABR and sound trial licking rates. An increase in hearing thresholds, as revealed by post-exposure ABR threshold shifts, could decrease the perceived intensity of sound trials and thus compromise perception of silent trials. Nevertheless, Experiment 1 showed that post-exposure day 0 threshold shifts were comparable between rats with lasting tinnitus behavior (tinnitus^(+)^) or transient tinnitus behavior (tinnitus^(-)^). Furthermore, hearing thresholds recovered by 7 weeks post-exposure, though tinnitus-like behavior persisted. Therefore, a threshold shift alone could not account for tinnitus behavior. This is supported by the consistency of sound trial licking rates over time, suggesting that hearing loss did not affect sound trial licking. If hearing loss had an effect, it likely would have resulted in decreased licking rates, given that lower intensity sound has been shown to yield lower activity rates [21, 22]. Consistent sound trial licking also indicated that the overall licking rate did not significantly fluctuate, and thus behavioral variability was not apt to account for tinnitus-like behavior. More detailed examination of auditory damage may be warranted in the future, to further explore its relationship with tinnitus and gauge the effect of other auditory confounds, like hyperacusis.

### Strengths of our behavioral paradigm

One strength of our paradigm is that animals can be trained within 16 training sessions (Figs 3 and 4), which translates to a little over 2 weeks. This is substantially shorter than the month or longer timeline required by some other paradigms, though such paradigms may possess their own advantages such as minimizing experimental discomfort [22] or enabling simultaneous electrophysiological recordings [20]. Admittedly, some paradigms require as little as a few days to a couple of weeks to train animals [19, 38, 53]. The tradeoff for shorter paradigms, however, can be a lack in some capabilities, such as detection of tinnitus pitch and distinction of onset versus lasting tinnitus status.

Another strength of our paradigm is that both onset and lasting tinnitus status, as well as their spectral properties, could be assessed within an individual animal without compiling data between testing sessions or across animals. This is an important distinction since many paradigms cannot determine, in the same animals, both onset and lasting tinnitus perception, tinnitus pitch, or when specific animals express tinnitus-like behavior [59]. The fact that tinnitus characteristics can also vary in humans [60, 61] emphasizes the need for animal paradigms that can effectively describe a range of characteristics. Thorough assessment of tinnitus development from an acute to chronic status may play a crucial role in pinpointing the fundamental mechanisms underlying tinnitus manifestation. If tinnitus cannot be identified in individual animals and if their tinnitus development cannot be followed over time or at specific times (i.e. requiring averaging of multiple testing sessions), then it is more difficult to target those specific mechanisms. That is, tinnitus etiology may vary between subjects and assessment time points. Detecting tinnitus pitch may be important as well, especially when examining tonotopically-organized structures in the peripheral and central auditory system. Therefore, pairing a versatile and reliable behavioral paradigm with methods such as chronic electrophysiological recordings, immunocytochemistry, and advanced neuroimaging modalities, may help significantly elucidate the mechanisms subserving tinnitus. Finally, since this paradigm works in rats, we anticipate that this behavioral paradigm may be further developed to assess behavioral evidence of tinnitus in other animals, including mice, gerbils and/or guinea pigs.

### Effects of other factors on the evaluation of tinnitus-like behavior

In addition to hearing threshold shifts and overall changes in licking, the potential effect of other factors, including confounds, must be considered. Changes to licking conditioning, for example, should be considered since rats in Experiment 1 were occasionally shocked for silent trial licks during post-noise-exposure testing sessions. Delivering footshocks during silent trials could condition tinnitus^(+)^ rats to reduce licking during narrowband sound trials that resembled their tinnitus frequencies. This form of conditioned suppression is in fact the basis for another behavioral paradigm [21, 24]. One difference in our paradigm, however, is that rats had to lick three times during sound trials to receive a water reward but never received footshocks for more than 50% of silent trial licks. As a result, tinnitus^(+)^ animals in our paradigm may have been less likely to suppress licking during sound or silent trials. Another distinction in our paradigm is that we placed greater emphasis on determining tinnitus behavior within individual animals, whereas noise-exposed animals are sometimes only analyzed as a group in the other paradigm. This is important since tinnitus development may vary across different animals and frequencies, and combining animal data may obscure tinnitus behavior. In one study that used a conditioned licking paradigm inspired by the Bauer and Brozoski method, individual animals were assessed [37]. Interestingly, several noise-exposed animals demonstrated higher suppression ratios, or greater licking, during silent trials compared to the mean sham suppression ratio. This suggests that rats may have been trying to lick during the silent trials due to tinnitus perception, which lines up with our present findings. This indicates that detecting tinnitus-like behavior can actually be similar between our paradigms, and that reconditioning is not necessarily confounding.

The effect of shock and conditioning changes were more directly explored in Experiment 2. Despite repeated tests with no footshocks and up to 4 weeks between tests, we could still identify noise-exposed rats that exhibited silent trial licking rates beyond the range of sham-exposed rats. This tinnitus-like behavior was observed in noise-exposed rats tested at 1-, 2-, 4-and 8-weeks post-exposure. These findings agreed with Experiment 1 in that more robust tinnitus perception was seen in a majority of noise-exposed animals shortly after noise exposure (Fig 9A). This is compared to later time points, where tinnitus perception appears to be less robust and only occurs in a portion of noise-exposed animals (Fig 9A, 9B and 9C). These findings also reflected Experiment 1 since noise-like tinnitus behavior tended to be most prominent, although some evidence of tonal tinnitus-like behavior was observed. Overall, these results suggest that post-noise-exposure footshocks and long intervals between testing sessions do not fundamentally change tinnitus behavior. It also shows the persistence of conditioned licking suppression attained in this behavioral paradigm, though there is a limit to the number of testing sessions that can be properly conducted without foot-shocks. Together, these findings cast doubt on alternate explanations for tinnitus-like behavior in Experiment 1 (i.e. changes in conditioning, random variability in post-exposure licking rates). Admittedly, silent trial licking was elevated compared to the post-exposure tests (with shocks) in Experiment 1, but distinctions between noise-exposed and sham-exposed rats could still be made. Given this, post-exposure footshocks may actually help improve detection of tinnitus by lowering the rate and variability of silent trial licking in non-tinnitus animals. This ultimately enables long-term testing of the development of onset and lasting tinnitus in individual animals, and may improve identification of tinnitus pitch.

Lastly, another factor that should be taken into account is hyperacusis, given its comorbidity with tinnitus [62, 63] and the presence of hyperacusis-like behavior in rodents [64]. Although hyperacusis behavior has not been considered via licking rates, it might influence licking rates during sound trials, given the altered auditory sensitivity in affected individuals. Since licking rates remained consistent in the present study, it is possible that our animals had hyperacusis but it did not affect sound trial licking, or that our animals simply did not have hyperacusis. One indication of hyperacusis-like behavior, however, is reaction-time [64], which we did not measure. To better evaluate the presence of hyperacusis and its interaction with tinnitus and other factors, reaction-time may be quantified in future studies.

## Supporting Information

S1 Fig Top and side views of the behavioral testing chamber. (CDR)

S2 Fig Illustrations showing phase 4 behavioral training during 6–8 and 10–12 kHz sound and silent trials. (XLSX)

S3 Fig Baseline rates of licking during silent trials (training sessions 11–16). (XLSX)

S4 Fig Baseline licking rates of eventual tinnitus^(+)^, tinnitus^(-)^, and control (ctrl) rats during different narrowband sound trials (A-E). (XLSX)

S5 Fig Licking rates over time for tinnitus^(+)^, tinnitus^(-)^, and control rats during silent trials (A-E). (XLSX)

S6 Fig Licking rates over time for tinnitus^(+)^, tinnitus^(-)^, and control rats during narrowband sound trials (A-E). (XLSX)

S7 Fig Licking rates during silent trials prior to injections (post-exposure week 8), as well as 3 hours and 5 days following saline (A) or salicylate (B) injections. (XLSX)

S8 Fig ABR hearing thresholds for tinnitus^(+)^, tinnitus^(-)^, and control rats prior to intense noise or sham exposure, and on post-exposure day 0 and post-exposure week 7. (XLSX)

S9 Fig Noise-exposed (Exposed) and sham-exposed (Sham) rats tested without footshocks at 1, 2, and 4 weeks post-noise-exposure. (XLSX)

S10 Fig Noise-exposed (Exposed) and sham-exposed (Sham) rats tested without footshocks at four weeks (A) and eight weeks (B) following exposure. (XLSX)
